# The abortion divide: Exploring the role of exclusion, loss of significance and identity in the radicalization process

**DOI:** 10.3389/fpsyg.2022.1025928

**Published:** 2022-11-30

**Authors:** Holly Knapton, Emma Renström, Magnus Lindén

**Affiliations:** ^1^Department of Psychology, Lund University, Lund, Sweden; ^2^Department of Psychology, Kristianstad University, Kristianstad, Sweden

**Keywords:** abortion rights, radicalization, social exclusion, significance loss, ingroup identity

## Abstract

**Introduction:**

Recently, the abortion issue has entered the spotlight in the USA, leading to potential radical actions. As the majority opinion on the abortion issue vary with state, some individuals will be in the numerical minority within their state, possibly evoking feelings of exclusion. Social exclusion can motivate a radicalization process. The aim of this paper is to explore how individuals in a numerical minority experience feelings of exclusion and significance loss and how this may drive radicalization in the context of the abortion issue.

**Methods:**

A quasi-experimental design was used and 534 respondents from naturally occurring numerical minority and majority groups based on state abortion opinion participated in an online survey.

**Results:**

Results showed that those in the numerical minority experienced exclusion and were more willing to engage in and endorse radical actions compared to those in the majority, regardless of position on the abortion issue. Serial mediation analysis revealed that the pathway between minority group status and engagement and endorsement of extreme actions was fully mediated by need-threat and ingroup identity.

**Discussion:**

Being in the numerical minority is associated with feelings of social exclusion, which may trigger a radicalization process. The results advance our understanding of when and who is vulnerable to radicalization and that social structures that perpetuate marginalization and inequality may contribute to radicalization. Results highlight the need to continue to explore radicalization from a group-based perspective and emphasize exploring mediating factors as a pathway from social experiences to willingness to engage with radical groups.

## Introduction

The purpose of this article is to explore how minority group status and associated feelings of exclusion may drive radicalization in the context of the abortion issue. In recent decades, following several high-profile terror attacks there has been an increased focus on understanding the radicalization process (Kruglanski and Fishman, 2009; King and Taylor, 2011). Prominent radicalization models argue that the radicalization process may function similarly across a variety of social, political, and religious issues (McCauley and Moskalenko, 2008; Doosje et al., 2016). The controversial topic of abortion rights in the USA is highly fraught with emotion and although much activism occurs within the boundaries of the law, it is a topic that is also associated with extremism, violence, and terrorism (Turell et al., 1990; Masucci, 2022). Within the past two decades, abortion providers have been murdered, abortion clinics bombed, death threats made to those seeking abortion services and intimidation tactics used (see Masucci, 2022 for overview). Recent changes in legislation have put the abortion debate into the spotlight and the increased focus has resulted in concerns that extremism and violent acts may increase from both sides of the abortion issue (Fox, 2022).

### Radicalization and social exclusion

Many radicalization models highlight social factors that may drive individuals to shift from socially accepted activism to more radical actions (McCauley and Moskalenko, 2008; Kruglanski et al., 2009, 2014; Kruglanski and Fishman, 2009; Doosje et al., 2016; Kruglanski et al., 2019, 2022). One such factor that has gained momentum in recent years is that of social exclusion and there is an increasing amount of empirical evidence that demonstrates the causal role that exclusion may have as a driver in the radicalization process (Knapton et al., 2015, 2022; Pfundmair, 2019; Renström et al., 2020; Pfundmair and Mahr, 2022). Being socially excluded leads to a loss of significance, which elicits a quest for significance and ways to restore it. The quest for significance radicalization model argues that this core motivation to maintain significance is a driver in the radicalization process (Kruglanski et al., 2009, 2014; Kruglanski and Fishman, 2009; Kruglanski et al., 2019). Belonging to a group can restore significance and recent research show that loss of significance increases subsequent extreme group identification (Bäck et al., 2018a; Renström et al., 2020). Recent studies also show that the link between significance loss and radicalization is mediated by identification with the ingroup (Knapton et al., 2022; Milla et al., 2022).

Much of the empirical research exploring the effects of exclusion has examined interpersonal exclusion (exclusion perceived due to a personal failing). However, recent research stress the importance of exploring exclusion from a group-level perspective (exclusion due to group membership), and this is particularly pertinent considering much of the radicalization literature deem radicalization a societal, group-based issue (McCauley and Moskalenko, 2008; Doosje et al., 2016; Knapton et al., 2022). Consequently, this paper aims to bring together traditional exclusion models and explore them at a societal level within a radicalization framework to provide an explanatory pathway of how minority group status, feelings of exclusion and threatened fundamental needs may drive individuals to identify with a radical ingroup and in turn be willing to participate in and endorse radical actions.

Radicalization is defined as the process in which an individual adopts extreme ideologies and beliefs, which may or may not result in extreme behavior (McCauley and Moskalenko, 2008). Recent models of radicalization are dynamic, exploring several pathways to extremism and the multifaceted factors that may drive individuals to engage (Borum, 2004; Horgan, 2008). Although many models have been proposed, and each has its unique contribution to the field, most models center around similar ideas trying to provide an explanation of how “ordinary” individuals may shift from normative behaviors to non-normative behaviors (McCauley and Moskalenko, 2008). This shift has been labeled the “conveyor belt.” This metaphor can be used to explain how individuals may slowly shift from normative activism to non-normative radical actions or even violence and exploring the early stages of this shift may be important in understanding how individuals escalate up the radicalization process (Moskalenko and McCauley, 2009). Thus, although radicalization is the process in which an individual may participate in violent actions, it is nuanced in level of severity and radicalization includes all non-normative action (any action that breaks social rules). Scholars consider non-normative and radical actions synonymous (Becker and Tausch, 2015). Consequently, these models all highlight the need to explore a normative population and how this population may be vulnerable to early radicalization rather than focusing to those already radicalized and at the violent end stage of the process (McCauley and Moskalenko, 2008).

The multifaceted pathway models give space not for one explanatory factor for radicalization but a dynamic interplay of several factors (McCauley and Moskalenko, 2017; Jensen et al., 2020). Nevertheless, it is possible to explore how individual factors may make individuals susceptible to radicalization pathways and one such theory is the quest for significance model. Kruglanski and Fishman (2009), Kruglanski et al. (2009, 2014)), Kruglanski et al. (2019) and Kruglanski et al. (2022) developed this motivational model of radicalization and it is a theory that is based on the idea that individuals have a need for recognition and positive self-esteem (Baumeister and Leary, 1995). However, certain events can happen in life that challenge this self-view, such as personal or societal grievances. When such events occur, it results of feelings of meaninglessness or humiliation and in turn results in significance loss. This loss of significance motivates radicalization, such that when an individual experiences significance loss they are motivated to regain significance and compensate for the loss. These compensatory activities will likely be conducted *via* their available social outlets, however if this fails, they may be drawn to extreme groups to fortify the basic need of significance. Adopting an extreme ideology and participating in extreme activities is one way to restore significance as these radical beliefs provide an individual with feelings of importance, meaningfulness, and control (Kruglanski et al., 2009, 2014; Kruglanski and Fishman, 2009; Kruglanski et al., 2019, 2022). Empirical research appears to support the quest for significance theory, with loss of social significance being a strong predictor of ideological crimes and evidence that it increases adherence to extremist ideas and participation in violent extremism (Webber et al., 2018; Jasko et al., 2020; Schumpe et al., 2020).

Significance can be lost in a variety of ways, but one way is *via* social exclusion (Kruglanski et al., 2019; Renström et al., 2020). Social exclusion is related to a host of negative outcomes. For instance, reduced mood and social pain, and in long-term cases, reduced life expectancy are some of the negative outcomes (Williams, 2007; Eisenberger, 2012; Rico-Uribe et al., 2018). The temporal need-threat model of ostracism explores the damage that occur from such an event on the individual’s fundamental needs (Williams, 2009). Specifically, this model argues that when an individual is excluded it depletes their fundamental needs such that individuals have a reduction in self-reported feelings of belonging, self-esteem, feelings of control and meaningful existence. The model argues that in response to exclusion, individuals will try to fortify these needs. For example, research show that individuals try to fortify these needs by regaining belonging *via* opportunities of inclusion. For instance, exclusion leads to increased attention to smiling faces in a crowd and compliance to a group and extra efforts in collaborative group tasks (Williams and Sommer, 1997; Carter-Sowell et al., 2008; Dewall et al., 2009). Given the desire to restore social needs and social connections following an episode of exclusion, it is not surprising that excluded individuals are more likely to be receptive to joining political groups (Bäck et al., 2015, 2018a, 2021; Knapton et al., 2015; Renström et al., 2021) and are more likely to participate in actions that conforms with the norm of the group (Dijker and Koomen, 2007; Bäck et al., 2013, 2018b).

In the radicalization literature, research also shows that marginalization and social exclusion seem to function to evoke a quest for significance. For instance, marginalized minority community members who feel a loss of significance are more likely to report increased support of fundamentalist groups, and recent experimental studies have linked social exclusion as a source of significance loss as a causal factor in individuals joining radical groups (Lyons-Padilla et al., 2015; Renström et al., 2020; Milla et al., 2022). Although there is a strong overlap in the need-fortification hypothesis and the quest for significance such that both are based on the need to restore fundamental human needs (Williams, 2007, 2009; Kruglanski et al., 2014; Knapton et al., 2015), there is very limited research exploring them in relation to one another (Renström et al., 2020). Consequently, it is essential to explore need-fortification within a radicalization framework.

### Group identification

When an individual is socially excluded from a group, they do not only lose a sense of belonging, but the identity associated with that group is threatened too. The rejection-identification model (Branscombe et al., 1999) details how discrimination and feelings of exclusion may result in individuals identifying more with their minority ingroup to protect against the negative outcomes of social exclusion. Research support this, with minority group members who experienced prejudice having increased minority ingroup identification (Verkuyten and Yildiz, 2007; Armenta and Hunt, 2009; Barlow et al., 2012; Cronin et al., 2012; Wiley et al., 2013). Identity is a key component when considering radicalization. Research indicates that ingroup identity to an extreme group is an important factor in determining how much an individual will endorse or engage in extreme actions (Hogg et al., 2010; Hogg and Blaylock, 2011; Hogg, 2014; Aghabi et al., 2017; Strindberg, 2020; Wagoner et al., 2021; Milla et al., 2022). Moreover, to restore status following significance loss, individuals may identify with an extreme group (Milla et al., 2022). Given the shifts in identity following an episode of exclusion, the pathway between an episode of exclusion, associated with significance loss, and radical actions will be mediated ingroup identification. Recent research support such a link with an experimental study revealing that those excluded due to their opinion on Brexit, showed increased identification with the EU and in turn increased willingness to join and participate in both normative and radical actions with a Pro-EU group (Knapton et al., 2022). Although there is extensive research exploring the phenomenon of social exclusion, most of the research has considered the exclusion or rejection of single individuals from a group, or at best, the exclusion of one small group by another. However, exclusion occurs at a societal level as well, with minority groups feeling excluded and marginalized within their society. Little research has explored what constitutes minority/majority groups in the exclusion context. Often, research exploring exclusion in minority/majority groups examines groups with a history of intergroup status and power differences (Branscombe et al., 1999; Barlow et al., 2012; Oxman-Martinez et al., 2012; Wiley et al., 2013). Thus, examining how majority/minority status based on political or social issue support may impact feelings of inclusion/exclusion, is necessary too. Specifically, it is important to understand if simply a difference in numerical status for a social/political cause in one’s social group is enough to trigger feelings of exclusion, or whether there needs to be a historical context of group power differences and inequality that are traditionally associated with studies exploring feelings of discrimination and exclusion. Hence, in this paper, minority/majority status is simply the numerical representation of opinions, and the extent to which this numerical status influences identification is an empirical question we explore. Given that recent research show that numerical representation of ones’ group within a context is enough to trigger feelings surrounding a sense of belonging (Glasford, 2021), we investigate if being in the numeric minority will trigger feelings of exclusion. We utilize the abortion issue in the US to explore if numerical minority is associated with feelings of exclusion and ultimately radicalization.

### The abortion issue

The abortion debate in the US has long been a controversial topic, with few social issues sparking more emotion than the discussion surrounding women’s reproductive rights (Turell et al., 1990). Although the abortion debate is a provocative topic in general, this highly sensitive issue was brought to the forefront of discussion when Texas introduced the “heartbeat bill” in August 2021, making abortion past the 6-week mark practically illegal, and reigniting the abortion debate in the US. The increased focus on the abortion debate is likely to increase salience of one’s stance on the issue and in turn as shown by previous studies, increase focus on one’s identification to one side of the issue (Hernández et al., 2021). With people’s opinions on a topic being more salient due to the issue being discussed, it is individuals may become acutely aware of the numerical majority opinion within their surroundings. Further, given that numerical representation of a group within a space can impact feelings of belonging (Glasford, 2021), it is likely that perceptions of numerical majority opinion surrounding an individual will impact feelings of exclusion or inclusion. Hence, individuals who are in the numerical minority due to their opinion on abortion may experience feelings of exclusion and be more willing to radically engage on behalf of an abortion activist group. Such feelings of exclusion should be associated with threatened fundamental needs of belonging, self-esteem, control, and meaningful existence (Williams, 2009; Glasford, 2021). Therefore, our first hypothesis is:

*H1*: Participants who are in the minority will have more threatened fundamental needs than participants in the majority.

Given that those in the numerical minority experience feelings of exclusion and that excluded individuals whose fundamental needs are threatened seek out ways to restore and fortify them and *via* increased willingness to join political groups (Williams, 2009; Knapton et al., 2015), our second hypothesis is:

*H2*: Participants in the minority will show increased willingness to participate in and endorse radical actions on behalf of a [Pro-life/Pro-choice] abortion activist group, than those in the majority.

**Figure 1:**
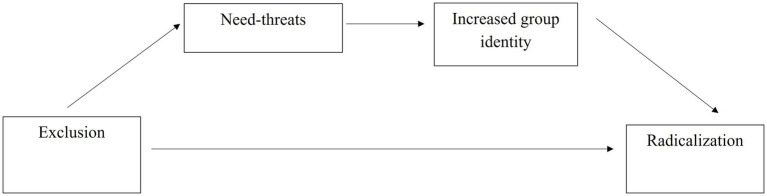
A conceptual model of the mediating pathway of need-threats and activist group identity between the effect of exclusion on willingness to participate and endorse radicalism.

Finally, identity is an important predictor in radical actions (Hogg et al., 2010; Hogg and Blaylock, 2011; Hogg, 2014; Aghabi et al., 2017; Strindberg, 2020; Wagoner et al., 2021; Milla et al., 2022). Given that increased ingroup identity may be used to buffer and fortify threatened needs following exclusion, those in the minority who experience threatened needs may identify more strongly with an activist group and in turn show increased willingness to participate in and endorse radical actions on behalf of an activist group (see conceptual model in Figure 1). Thus, our final hypothesis is:

*H3*: The effect of minority group status on willingness to participate and endorse radicalism is mediated by increased threatened needs and in turn increased identification with the ingroup.

## Methods and data

### Research design

In this study we used a quasi-experimental design where naturally occurring groups were examined. We recruited Pro-life and Pro-choice participants from Pro-life and Pro-choice states. When a participant’s opinion on abortion matched the majority opinion of the state they lived in (e.g., Pro-life supporter living in a Pro-life state) they were in the majority and conversely when the participant’s opinion differed from the majority opinion of the state (e.g., Pro-choice supporter living in Pro-life state) they lived in they were in the minority. As a result, we ended up with 2 groups that formed the independent variable numerical status (minority/majority). The dependent variables analyzed in this paper are *Willingness to participate in radical actions* and *endorsement of radical actions*. There were two mediator variables; *activist group identity,* and *Need-threats.* The study was set up on *Qualtrics.*

### Participants

A sample of 543 participants was recruited from the online study platform *Prolific Academic*. *Prolific Academic* is an online recruitment website with the purpose of advertising open research studies to participants. To be eligible, participants had to be American citizens currently live in the USA. Pro-life and Pro-choice participants were recruited from both Pro-life and Pro-choice states using the prescreening criteria available on *Prolific Academic*. Participants responded to the prescreening criteria when joining the website and self-identified as either Pro-choice, Pro-life or Do not want to answer. Using data from a public opinion survey, the participants were selected from the top 6 most Pro-life states and Pro-choice states (Diamant and Sandstrom, 2020). Pro-life states were Arkansas, Mississippi, Alabama, West Virginia, Louisiana and Kentucky. The Pro-choice states were Massachusetts, Vermont, Connecticut, New Hampshire, Rhode Island and New York.

Participants were naturally based in one of the two conditions: majority or minority. In the *majority* condition, there was 281 participants (mean age of 37.29, SD = 13.81) of which 129 were men, 142 women and 3 other. In the *minority* condition there was 260 participants (mean age of 36.90, SD = 13.82) of which 128 were men, 132 women and 2 other. Participants were rewarded £2.10 for their participation.

### Procedure and measures

On starting the survey participants were told this was an online survey exploring their thoughts and feelings on abortion, reproductive rights, and abortion legislation. Further, they were told that the final section of the questionnaire would contain questions from a third-party group. This group was fictional. After reading the survey information in which the participant was informed of their right to withdraw and that their data would be completely confidential, they provided consent to participate.

Following the information, the participants were asked several questions about their thoughts and feelings regarding abortion and about the perception of abortion opinions in their state. Participants were prompted with the phrase “Given that a **majority** in your state are [Pro-life/Pro-choice] please describe how this makes you feel” and this was adjusted depending on whether the participant lived in a state that was majority Pro-life or Pro-choice. Participants were then presented with an adapted form of the need-threat scale formed of 20 items (Williams, 2009). Examples of these items are shown in Appendix A. Participants responded on a 5-point scale with 1 indicating *not at all* and 5 indicating *extremely.* The 20 items were combined and averaged to give a total need-threat score. The Cronbach’s alpha was good, *α* = 0.66.

In the next section, participants were informed that the following section was a survey by a third-party group. This group was fictional but presented as real to the participants. Participants were told that the answers to the survey prior and the answers to the third-party group would be examined separately and we apologized for any overlap in questions. This statement aimed to make the third-party group survey more believable. Once participants clicked “continue” they were presented with a brief description of the group. The group differed based on whether the participants identified as Pro-life or Pro-choice, with the group presented designed to be congruent with the participants’ opinion on abortion. Hence, if the participant identified as Pro-life, they were presented with the group “Pro-life for America” and if they identified as Pro-choice, they were presented with the group “Pro-choice for America.” The group descriptions were made as similar as possible in tone and phrasing, and differed only in content to match the abortion position (e.g., anti-abortion sentiments for the Pro-life group and freedom of choice in reproductive decisions in the Pro-choice group). At the end of the group descriptions, both groups explained that they were interested in recruiting new members and wanted to know what actions appealed to possible new members. After this statement, several identity and participation items followed. Participants were asked about their identification with the activist group, which consisted of 3 items: “I feel I could identify with [Pro-life/Pro-choice] for America”; “I feel I could connect with other members of [Pro-life/Pro-choice] for America” and “I identify with the aims of [Pro-life/Pro-choice] for America.” Participants responded on a 7-point scale from 1 = *strongly disagree* to 7 = *strongly agree.* The items were combined and averaged to form an activist group identification index (*α* = 0.93).

Following this, participants were asked about willingness to engage in radical action. They were asked how willing they would be to participate in the 3 following forms of non-normative collective action on behalf of the group: take part in an occupation, vandalize buildings, and protest on social media (e.g., post offensive material on opposing groups’ social media). Participants responded on a 5-point scale from 1 = *not at all willing* to 5 = *very willing*. The 3 items were combined and a willingness to engage in radical action index formed (*α* = 0.66).

Finally, participants were asked about endorsement of radical actions. This was formed of two items: “I think even extreme methods are justified and acceptable to reach the goal of a greater American society. That is, a combination of traditional methods like petitions, but also direct actions that may extend beyond the borders of the law” and “I think most [Pro-life/Pro-choice] supporters in society agree that extreme methods are justified and acceptable to reach the goal of a [Pro-life/Pro-choice] for America for a better American society.” Participants indicated on a 7-poing Likert scale how much they agreed with the statement, with 1 indicating they *strongly disagreed* and 7 indicating they *strongly agreed*. The two items were combined, and an endorsement of radical actions index formed (*r* = 0.66).

This marked the end of the study and participants were debriefed, thanked for their time, and given the opportunity to provide any feedback/questions they had.

## Results

### Preliminary analyses

Because we wanted to explore natural inclusion/exclusion based on whether the participant’s opinions matched or mismatched the majority of the state, we first analyzed if participants perceived the same state majority as our pre-set states (Diamant and Sandstrom, 2020). Participants rated the perceived percentage of Pro-life/Pro-choice supporters in their state. A *t*-test was conducted in which our classification of states being Pro-life or Pro-choice was entered as an independent variable and the participants’ perception of percentage of Pro-life/Pro-choice supporters in their state was entered as a dependent variable. The t-tests revealed that participants significantly, *t*(508) = −13.24, *p* < 0.001, Cohen’s *D* = 1.18, rated a higher percentage of Pro-life supporters (*M* = 71.33, SD = 14.86) living in Pro-life states, compared to Pro-choice supporters (*M* = 49.71, SD = 21.12), and a significantly, *t*(508) = 16.89, *p* < 0.001, Cohen’s *D* = 1.51, higher percentage of Pro-choice supporters (*M* = 57.90, SD = 20.75) living in Pro-choice states compared to Pro-life supporters (*M* = 29.97, SD = 16.00). Thus, the analyses confirmed that participants’ perceptions of the majority abortion stance of the state matched that of previous research and thus what we based on categorization of Pro-life/Pro-choice states on (Diamant and Sandstrom, 2020).

### Main analyses

To test our hypotheses a series of t-tests were conducted. The descriptive statistics and correlations for all variables are presented in Table 1.

**Table 1:** Means, standard deviations, and correlations for all variables.

	Mean	SD	Need-threats	Activist identity	Radical Actions
Need-threats	2.57	0.44			
Activist group identity	5.86	2.47	0.28**		
Radical actions	2.10	0.91	0.24**	0.53**	
Endorsement of radical actions	2.83	1.66	0.17**	0.19**	0.46**

**p < 0.01, *p < 0.05.

The first hypothesis stated that *participants who are in the minority will have more threatened social needs than participants in the majority.* The first t-test was conducted with minority/majority as the independent variable and the need threat index as dependent variable. There was a significant effect of minority/majority on the need threat scale, *t*(521) = −2.82, *p* = 0.005, Cohen’s *D* = 0.23 such that those in the minority had significantly higher threatened fundamental needs (*M* = 2.62, SD = 0.43), than those who in the majority (*M* = 2.51, SD = 0.44). Thus, hypothesis 1 was supported—simply being in the numerical minority is associated with feelings of being excluded.

The second hypothesis stated that *participants in the minority will show increased willingness to participate in and endorse radical actions than those in the majority*. As a result, two *t*-tests were conducted to explore the effect of minority/majority group status on willingness to participate in radical actions and endorsement of extreme actions. The results revealed a significant difference, *t*(531) = −2.21, *p* = 0.027, Cohen’s *D* = 0.19, such that those in the minority (*M* = 2.15, SD = 0.92) were significantly more willing to participate in radical actions than those in the majority (*M* = 1.97, SD = 0.90). There was no significant effect of minority status on endorsement of radical actions. Consequently, hypothesis 2 was partially supported.

### Mediation analysis

**Figure 2:**
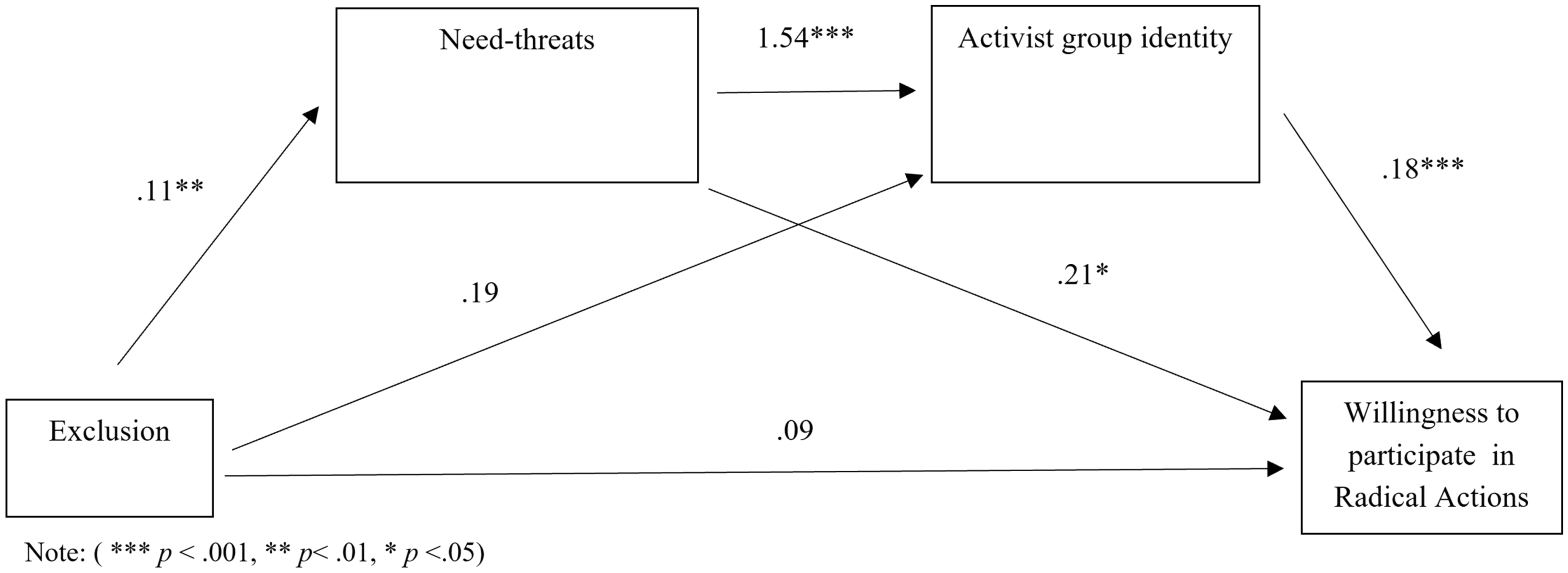
Serial mediation model with coefficients for pathway between exclusion and willingness to participate in radical actions with mediators need-threats and activist group identity.

**Figure 3:**
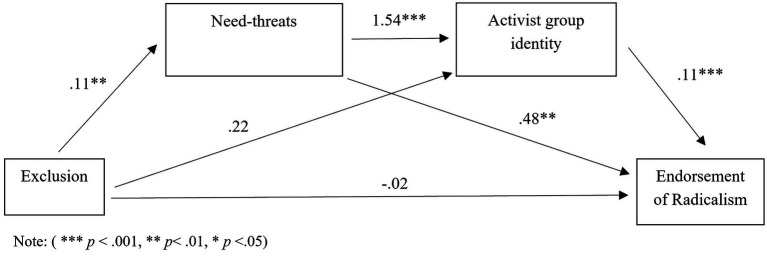
Serial mediation model with coefficients for pathway between exclusion and endorsement of radical actions with mediators need-threats and activist group identity.

Hypothesis 3 stated that *the effect minority group status on willingness to participate and endorse radicalism is mediated by increased threatened needs and in turn increased identification with the ingroup.* A serial mediation analysis was conducted using Model 6 in the SPSS macro PROCESS (Hayes, 2013). Using bias corrected bootstrapping with 95% confidence intervals with 5,000 bootstrapping samples, the indirect effect of minority group status on willingness to participate in radical actions through both threatened fundamental needs and activist group identity was conducted. As a result, need-threat and activist group identity were added as mediators between the predictor variable (minority/majority) and the outcome variable, willingness to participate in radical actions. Effects were significant if the 95% confidence intervals associated with each analysis did not include 0. As can be seen in Figure 2, there were multiple significant pathways, but the sequential mediation analysis revealed a significant indirect pathway from minority group status *via* need threat and activist group identity to willingness to participate in radical actions was significant when all pathways were considered (see Table 2). Minority group members who had higher threatened needs showed higher activist group identity and were more willing to participate in radical actions with the activist group. This analysis was re-run using the dependent variable endorsement of radical actions and the results again revealed a significant indirect pathway from minority group membership to endorsement of extreme actions *via* need-threat and group identity (see Figure 3; Table 2). This finding mirrors that found for willingness to participate in radical actions. Consequently, hypothesis 3 was fully supported.

**Table 2:** Direct and indirect effects of exclusion on willingness to participate in radical actions and endorsement of radicalism from bootstrapping with confidence intervals in parenthesis.

	Radical actions	Endorsement of radical actions
Direct effects	0.08 (−0.05; 0.22)	−0.02 (−0.30; 0.26)
Indirect effects		
Exclusion – Need-threats– Radicalism	0.02 (0.00; 0.05)	(0.01; 0.11)
Exclusion – Group Identity–Radicalism	0.03 (−0.04; 0.11)	(−0.02;0.08)
Exclusion – Need-threats– Group Identity–Radicalism	0.03 (0.01; 0.06)	(0.01; 0.04)

Level of confidence for all confidence intervals is 95%. Results are based on 5,000 bootstrap samples.

## Discussion

The focus of this article was to expand the understanding of the radicalization process by exploring the role of minority/majority group status, feelings of exclusion, and identity on willingness to participate in and endorse radical actions on behalf of an abortion activist group. We provide an empirical test of the significance loss hypothesis (Kruglanski et al., 2014, 2022) and illuminate how numerical status and representation (e.g., minority/majority group) could be a starting point in a radicalization process. The study was situated using the significance quest model of radicalization as a framework for explaining the motivational factors that drive an individual to be receptive to extremist groups (Kruglanski and Fishman, 2009; Kruglanski et al., 2009, 2014; Kruglanski et al., 2019). Significance loss can be triggered by social exclusion and we wanted to explore if feelings of exclusion could lead to radicalization (Kruglanski et al., 2014). Previous research has demonstrated that belongingness concerns occur among individuals who are in the numerical minority within a group, and we posited that being a minority group member would trigger feelings of exclusion and thus significance loss which would motivate individuals to radicalize (Glasford, 2021). Our findings supported this notion, showing that individuals in the numerical minority experienced feelings of exclusion as measured by need-threats (Williams, 2009). Further, minority group status significantly predicted willingness to engage in radical actions, and further this effect was mediated by both need-threat and ingroup identity. Our findings thus provide insight into the exclusion literature and the radicalization literature, and these contributions are discussed below.

### Theoretical contributions

#### Group composition and feelings of exclusion

A fundamental feature of the study was that we assumed that being in the numerical minority would threaten the fundamental needs as theorized by the temporal need-threat model (Williams, 2009). This assumption was based previous research that has showed that being in the numerical minority impacts feelings of belonging (Glasford, 2021). Our findings supported this assumption. Individuals in the numerical minority (Pro-life supporter living in Pro-choice state, or Pro-choice supporter in a Pro-life state) had significantly higher threatened needs than individuals in the majority (e.g., Pro-life supporter living in Pro-life state). Hence, it can be argued that minority group membership triggers feelings of exclusion. However, this study was conducted in a time when abortion rights were highly discussed, so these findings can only be considered in a context where the cause of exclusion is currently salient. Specifically, in this case, the increased focus on abortion rights following the changes in legislation may make membership to the group (Pro-life/Pro-choice) more pertinent. This conclusion is supported by research showing that identities can become more prominent when a contextual factor makes them salient (Hernández et al., 2021). Thus, focus on group membership due to increasing media and societal attention to abortion rights, may make the effect of minority/majority membership on feelings of exclusion (threatened needs) more prominent. Consequently, in this unique context it can be determined that those in the numerical minority on their abortion stance were feeling societally excluded as demonstrated by their threatened needs. Future research may want to replicate this study and explore if this finding is replicated when an individuals’ identity is not salient to explore if numerical group status impacts feelings of exclusion in a similar manner. Nevertheless, the findings confirm previous research that has shown that simple numerical distribution of group members can result in feelings of exclusion (Richman et al., 2011; Glasford, 2021) and to our knowledge it is the first study to demonstrate that this form of exclusion also has the ability to threaten an individuals’ fundamental needs as outlined in the temporal need-threat model (Williams, 2009).

#### Exclusion and radicalization

Given that feelings of exclusion were established in the minority, in line with previous research it was likely that minority individuals would try to establish social connections to fortify their threatened fundamental needs as seen in traditional social exclusion research (Williams, 2007, 2009). In line with previous empirical studies that have demonstrated the causal role that exclusion has with engagement in political and radical actions, we argued that those in the minority would be more willing to participate in and endorse radical actions (Pfundmair, 2019; Renström et al., 2020; Knapton et al., 2022). Our findings confirmed this. The results showed that those in the minority were significantly more willing to participate in radical actions compared to those in the majority. However, there was not a significant effect of minority/majority group status on endorsement of radical actions. Nevertheless, the findings confirmed previous studies that used interpersonal or small group-based exclusion to demonstrate that exclusion can lead to increased willingness to participate when the exclusion occurs at a societal level. This is not surprising given that our findings revealed that those in the minority have reduced fundamental needs, and there is extensive documented research that has revealed the efforts that individuals go to try and restore these needs, including joining radical groups (Williams, 2007, 2009; Knapton et al., 2015; Renström et al., 2020). As such, the study adds to the both the social exclusion literature but also confirms the motivational mechanism to restore significance following the loss of significance due to exclusion as outlined in the quest for significance radicalization model (Kruglanski et al., 2014, 2019). This is an important contribution as there is little empirical evidence that has tested the significance loss model and this confirms the experimental research that has been conducted (Bélanger et al., 2019; Renström et al., 2020).

#### Identity and radicalization

An important part of our research was to establish an explanatory pathway between exclusion and radical actions. There is extensive research indicating that exclusion can impact identity levels. For instance, feelings of discrimination, exclusion and loss of significance all increase ingroup identity (Branscombe et al., 1999; Verkuyten and Yildiz, 2007; Armenta and Hunt, 2009; Barlow et al., 2012; Cronin et al., 2012; Wiley et al., 2013; Knapton et al., 2022; Milla et al., 2022). Arguably, this is to protect fundamental needs, buffer against the negative effects of exclusion and benefit from the positive status or belonging associated with a collective identity to maintain needs and a feeling of significance (Branscombe et al., 1999; Milla et al., 2022). Given that ingroup identity is an established factor in radical actions, the shift in identity may be a driving factor in the causal link between exclusion and radical actions (Hogg et al., 2010; 2012; Hogg, 2014; Aghabi et al., 2017; Strindberg, 2020; Wagoner et al., 2021; Milla et al., 2022). Thus, we proposed that those in the minority, with threatened fundamental needs would be the most willing to identify with a radical group and in turn most willing to participate in radical actions. As a result, a serial mediation model was run examining the pathway between minority group status and increasing willingness to participate in radical actions and endorsement of radical actions *via* threatened needs and identity. The findings revealed a significant indirect pathway for both dependent variables. Minority individuals who had higher threatened needs showed higher identification with the activist group, which increased willingness to participate in radical actions and to endorse radical actions. Thus, although no main effect of minority group on endorsement of radicalism was found, there was a significant indirect effect *via* need-threat and ingroup identity.

Our findings not only provide insight to a pathway between exclusion and radical actions but also add to the understanding of the rejection-identification model within the backdrop of the temporal need-threat model (Branscombe et al., 1999; Williams, 2009). The findings of the study suggest that a desire to fortify threatened needs may be driving increased identification with an accepting minority group, given that our findings show that it was those with higher threatened social needs who had higher identification with the activist group. Moreover, the study demonstrates that the rejection-identification model can be applied to other contexts than ethnic based discrimination, which has been the focus of most previous research on this model (Verkuyten and Yildiz, 2007; Armenta and Hunt, 2009; Barlow et al., 2012; Cronin et al., 2012; Wiley et al., 2013). The present study highlights that perceived exclusion at a societal level may be due to ones’ political beliefs or personal values or simply through the perception of a numerical majority. This is an important contribution given that much of the focus into societal level exclusion explores groups with historical intergroup power differences, whereby the exclusion is examined in relation to occurring a dominant ethnic (often White) majority (Verkuyten and Yildiz, 2007; Armenta and Hunt, 2009; Barlow et al., 2012; Cronin et al., 2012; Wiley et al., 2013). Given research that shows power and status differences in groups can trigger a desire for social change, the effects seen in this study may be stronger in groups with a discrepancy in power and status (Spears et al., 2001; Scheepers et al., 2006). Thus, future research may want to consider replicating this study in other intergroup contexts.

Finally, and arguably most importantly, this study adds to the understanding of the way in which social exclusion may drive willingness to engage in radical activism through a mechanism of identity and provides further support to the existing literature that highlights the important role that identity has regarding individuals’ political participation and radical actions (Klandermans et al., 2002; van Zomeren et al., 2008; Borum, 2011; Knapton et al., 2022). The study confirms recent findings demonstrating a link between exclusion, ingroup identity and participation in normative and radical actions (Knapton et al., 2022) but also adds to it by highlighting the role that threatened fundamental needs have regarding driving increased identification. The findings bring together the temporal need-threat model, rejection identification model and the literature exploring social exclusion as a driver of radicalism to provide an explanatory pathway for the link between exclusion, identity, and radical engagement (Branscombe et al., 1999; Williams, 2009). As such, the findings also add to current radicalization models by providing empirical evidence that help encompass several factors, such as loss of significance, belonging, identity, marginalization, societal grievances, all of which are noted in multiple models as driving mechanisms (Moghaddam, 2005; McCauley and Moskalenko, 2008, 2017; Kruglanski et al., 2009, 2014; Borum, 2011).

#### Methodological contributions

Another important aspect of our study was to explore societal level exclusion using a quasi-experimental design. Much of the radicalization literature highlights radicalization to be a group-based, societal level concept but there is very little empirical evidence in the exclusion literature that has explored the phenomenon in this manner (McCauley and Moskalenko, 2008; Kruglanski et al., 2009, 2014; Kruglanski and Fishman, 2009; Doosje et al., 2016). Given evidence that numerical distribution of a group function as determining minority/majority status and that this can lead to feelings surrounding belongingness, we used the unique geographic divide of abortion opinion in the USA to conduct a quasi-experimental study to explore minority/majority group status as a driver of feelings of exclusion (Glasford, 2021). Participants living in states that matched their opinion (e. g. Pro-life supporter living in Pro-life state) were in the majority and participants living in states incongruent with their personal opinion (Pro-life supporter living in Pro-choice state) were in the minority. Measures of feelings of exclusion using the need-threat scale were conducted and these in turn were used to explore how feelings of belonging due to group membership (minority/majority) can drive willingness to engage in radical actions *via* ingroup identity. Many exclusion paradigms have looked at interpersonal or smaller group- based exclusion, but none have examined the impact on these fundamental needs at a societal level (Williams, 2007). Further, much of the societal level exploration of exclusion has examined exclusion due to ethnicity, and this further confirms previous research that feelings of exclusion can occur due to other factors than ethnic or cultural conflicts, such as in this case, one’s opinion on a social issue (Branscombe et al., 1999; Bélanger et al., 2019). Consequently, this study contributes to our methodological understanding of how to explore both exclusion and radicalization and provides support for using quasi-experimental designs to investigate societal level issues in a causal manner and help explain phenomena using mechanisms traditionally explored solely within a controlled experimental context.

### Limitations

Although we did not test significance loss directly, there is considerable overlap in features between the significance loss model and the temporal need-threat model, which both state that following an episode of social exclusion individuals will be more receptive and willing to join an extremist group as a way of fortifying belonging and significance (Williams, 2007, 2009; Kruglanski et al., 2014). We use the terms interchangeably such that in line with previous research we assume social exclusion will trigger a loss of significance and motivate radicalization though both a desire to fortify fundamental needs and regain significance (Renström et al., 2020).

One of the limitations surrounds the assumption that those in the minority would be socially excluded. Given that this was a quasi-experiment using naturally occurring groups, there was little control in the extent to which individuals felt excluded/included. Based on the measure of need-threats as used in previous exclusion studies, our results mirrored various controlled, manipulated episodes of exclusion, in that those individuals who were excluded (the minority) in our study showed threatened fundamental needs, compared to those who were included (the majority; Eisenberger et al., 2003; Smith and Williams, 2004; Zadro et al., 2005; Williams, 2007; Carter-Sowell et al., 2008; Knapton et al., 2015). However, this measure was in relation to how they felt regarding their states, and what we can be unsure of is if there are “microcommunities” within those states where likeminded individuals happen to live near on another or if an individual surrounds themselves with friends who are like minded even if they are not physically present in which they feel a sense of belongingness. Nevertheless, given that minority individuals showed threatened needs similar to when exclusion is controlled and manipulated experimentally, the ecological validity this study outweighs any operationalization concerns. Yet, future studies that may use a similar quasi-experimental set up may want to consider ways in which to measure feelings of exclusion in such a context independent of the need-threat measure.

In line with the above, it is important to consider that exclusion is multifaceted. Minority groups who are excluded from society often experience other factors such as lower socio-economic levels or other factors that may put them at a disadvantage (Schmitt and Branscombe, 2002; Stuart et al., 2020). In our study, we did not measure for such socio-economic factors as a confounding variable. Given the sample used was across states and across abortion opinion, it was not expected that there would be a significance difference across socio-economic factors of the naturally occurring groups but future research using a similar research design should consider measuring these variables too.

Another limitation is the lack of a significant finding with regards to the effect of minority group status on endorsement of extreme actions. Although an indirect effect was found *via* a pathway of need-threat and identity, a main effect was expected. A possible reason for this is that we argued that feelings of exclusion as triggered by being a member of a minority group would drive an individual to participate and endorse radical actions due to a need to desire reconnection to restore belongingness needs. A possible reason therefore why we found no effect of minority group status on endorsement of actions is that simply endorsing an action has a minimal social element, compared to participating in radical actions with a group. Thus, it may only be those who identified highly with the group that also then endorsed the actions given that higher ingroup identification is linked to higher endorsement of violent ideology (Milla et al., 2022).

Finally, we examined radical action intentions rather than actual radical action participation. Thus, caution should be taken regarding how much these intentions would reflect real life behavior. Future research should consider asking about previous engagement.

## Implications and conclusion

The social exclusion literature has clearly demonstrated the role of exclusion regarding driving political engagement (Bäck et al., 2015, 2021; Knapton et al., 2015; Bäck et al., 2018a; Renström et al., 2020). However, what is the mechanism explaining this link and its role in radical actions and radicalization is less examined although highly discussed from a theoretical and/or qualitative perspective. The quest for significance model of radicalization highlights that an episode of social exclusion may motivate someone to regain their significance *via* radical groups and the temporal need-threat model explains willingness to engage in radicalism *via* a desire to fortify one’s fundamental needs (Williams, 2009; Kruglanski et al., 2014). In this paper, these theories are used in unison and along with previous research and theory assume that an episode of exclusion drives a quest for significance *via* the threatened fundamental needs. The findings of our paper support both theories, showing that feelings of exclusion drive willingness to participate in and endorse radical actions, and further that this pathway is mediated by this need to restore needs and significance. However, it also contributes to the literature by demonstrating the role that identity plays, indicating individuals with threatened needs seek out accepting identities and readily identify with an accepting ingroup. As a result, this paper brings together the temporal need-threat model and the rejection identification model in the context of radicalization and uses them as a explanatory pathway for the effect of social exclusion on radical actions. This paper adds to the social exclusion literature by exploring exclusion from a societal perspective in a quasi-experimental way and it helps pave the way for future research to consider exploring naturally excluded groups. It emphasizes that exclusion can be perceived from sheer numerical distribution of group members in a setting and further confirms that even subtle exclusion cues can cause reduced well-being (Gaertner et al., 2008; Wirth et al., 2010; Glasford, 2021) The findings highlight the importance of continuing to explore the nuances in social exclusion from varying intergroup levels and highlights the need to continue to explore the driving role of identity in activism engagement and radicalization.

Finally, there are concerns given the recent changes that there may be an upsurge in violence from both sides of the abortion debate, and these research highlights that this may be likely if individuals continue to feel excluded and isolated in their communities due to their opinion. Abortion is a provocative issue that is surrounded with emotion and possible conflict. This research highlights the need for individuals’ opinions to be seen and heard, and for an inclusive community to be fostered to prevent individuals becoming radicalized.

## Data availability statement

The raw data supporting the conclusions of this article will be made available by the authors, without undue reservation.

## Ethics statement

The studies involving human participants were reviewed and approved by Swedish Ethical Review Authority. The patients/participants provided their written informed consent to participate in this study.

## Author contributions

HK, ER, and ML all contributed to conception and design of the study. HK performed the statistical analysis and wrote the first draft of the manuscript. ER and ML provided valuable input for important revisions. All authors contributed to the article and approved the submitted version.

## Funding

Funding for data collection and open access publication fees were provided by Lund University.

## Conflict of interest

The authors declare that the research was conducted in the absence of any commercial or financial relationships that could be construed as a potential conflict of interest.

## Publisher’s note

All claims expressed in this article are solely those of the authors and do not necessarily represent those of their affiliated organizations, or those of the publisher, the editors and the reviewers. Any product that may be evaluated in this article, or claim that may be made by its manufacturer, is not guaranteed or endorsed by the publisher.

## Appendix A

Given that a **majority** in your state are [**Pro-life/Pro-choice]** you feel (Participants responded on a 5-point scale with 1 indicating *not at all* and 5 indicating *extremely*.):

**Table:**

I feel”disconnected”
I feel rejected
I feel like an outsider
I feel I belong in [STATE OF RESIDENCE]
I feel others interact with me a lot
I feel good about myself
My self-esteem is high
I feel liked
I feel insecure
I feel satisfied
I feel invisible
I feel meaningless
I feel non-existent
I feel important
I feel useful
I feel ashamed
I feel humiliated
I feel hopeless
I feel angry
I feel powerful
I feel I have control over the course of events
I feel I have the ability to significantly alter events
I feel I am unable to influence the action of others
I feel others decided everything
